# Compact transcriptional activators: Therapeutic gene regulation in ocular disease

**DOI:** 10.4103/NRR.NRR-D-25-01001

**Published:** 2026-02-05

**Authors:** Zhiquan Liu, Lauren Y. Chuu, Siyu Chen, Yang Sun

**Affiliations:** Department of Ophthalmology, Stanford University School of Medicine, Palo Alto, CA, USA; University of California, Berkeley, CA, USA

Vision loss from ocular diseases affects millions worldwide and represents a significant challenge to global health. One of these conditions, retinitis pigmentosa (RP), comprises a heterogeneous group of inherited retinal disorders caused by mutations in a wide array of genes. Although substantial progress has been made in elucidating the molecular causes of RP and related inherited retinal diseases, effective therapies remain limited, particularly for monogenic inherited forms (Tsang and Sharma, 2018). The clinical development of several adeno-associated virus (AAV)-based gene augmentation therapies is advancing; some have gained regulatory approval, including Luxturna for *RPE65*-associated retinal dystrophy (Ng et al., 2024). The eye presents several advantages as a target organ for gene therapy; it is immune-privileged, anatomically accessible, and amenable to longitudinal monitoring through imaging and functional testing (Ng et al., 2024). These features position the eye as a model system for implementing novel genetic therapies, including gene editing and transcriptional modulation. However, current gene augmentation may be limited by the lack of regulatory elements. To broaden therapeutic strategies beyond traditional gene augmentation, especially for conditions driven by gene loss-of-function or haploinsufficiency, innovative approaches such as endogenous gene activation are urgently needed.

Recent advances in CRISPR-based transcriptional activators (CRISPRa) have opened new avenues for treating genetic diseases by enabling targeted upregulation of endogenous gene expression. Unlike gene augmentation strategies that rely on exogenous cDNA delivery, CRISPRa allows transcriptional activation of native genes within their chromosomal context. This approach preserves the endogenous regulatory architecture and spatiotemporal expression patterns. CRISPRa is well suited for haploinsufficiency and other loss-of-function conditions where partial recovery of activity (e.g., enzyme) may be sufficient for therapeutic benefit. Furthermore, CRISPRa systems offer the potential for activation of multiple genes, which is advantageous given the heterogeneous nature of inherited retinal disorders.

Because they typically consist of a catalytically dead Cas9 protein fused to transcriptional activation domains such as VP64, p65, and Rta (VPR), CRISPRa systems enable programmable gene activation without inducing double-stranded DNA breaks (Ng et al., 2024). Because enzymatically deficient Cas, coupled to the proper guide RNA, can efficiently target transcriptional machinery to the genes of interest, CRISPRa bypasses the limitations of traditional gene augmentation, which often relies on the exogenous overexpression of therapeutic genes via AAV vectors. CRISPRa allows precise activation of native gene loci, potentially resulting in physiologically relevant expression patterns and improved long-term safety.

A key limitation of current CRISPRa systems is the challenge of efficient *in vivo* delivery. Most widely used activator platforms are based on Streptococcus pyogenes Cas9 (SpCas9, 1368 amino acids), whose large size exceeds the packaging capacity of a single AAV vector. As a result, dual-vector strategies are often required, which complicate delivery logistics and reduce therapeutic efficiency (Ng et al., 2024). The development of compact CRISPRa tools is critical for overcoming this barrier and treating disorders such as RP, where the target retina is anatomically constrained. Xu et al. (2021) introduced dCasMINI, a compact CRISPRa system comprising only 529 amino acids—less than half the size of SpCas9. While this system demonstrated robust transcriptional activation *in vitro*, its potential for *in vivo* gene regulation and therapeutic application is currently being actively studied.

**Figure 1:**
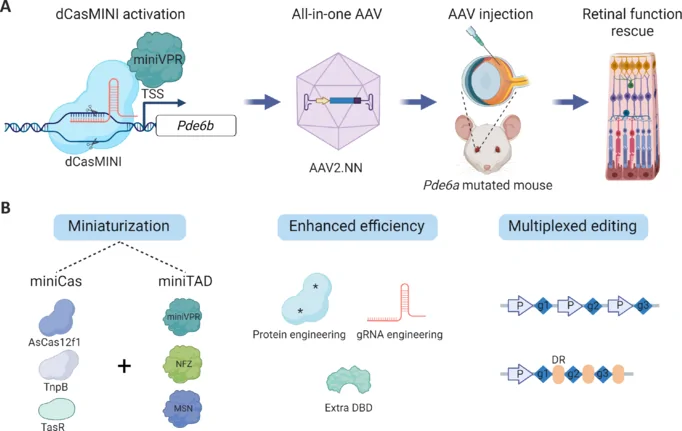
Schematic overview of dCasMINI-mediated CRISPRa therapy and future optimization strategies. (A) Schematic of an all-in-one AAV2.NN vector delivering a dCasMINI-miniVPR system targeting the *Pde6b* promoter. Upon intravitreal injection into *Pde6a*-mutant mice, the system activates *Pde6b* transcription, improving retinal structure and visual function. (B) Future directions for optimizing CRISPRa-based gene therapy. Miniaturization: smaller Cas proteins (e.g., AsCas12f1, TnpB, and TasR) and compact transcriptional activation domains (miniTADs such as miniVPR, NFZ, and MSN) may reduce vector size. Enhanced efficiency: improvements can be achieved through protein engineering, optimized gRNA design, and the addition of extra DBDs. Multiplexed editing: strategies such as tandem gRNA expression with individual promoters or self-cleaving RNA elements (e.g., tRNA and ribozymes) can enable multi-gene activation within AAV size constraints. Created with BioRender.com. AAV2.NN: An engineered AAV2 serotype; DBD: DNA-binding domain; DR: direct repeat; miniCas: miniaturized CRISPR-associated protein; miniTAD: miniaturized transcriptional activation domains; miniVPR: miniaturized VP64–p65–Rta; MSN: MRTF-A, STAT1 and eNRF2 domains; NFZ: NCOA3, FOXO3 and ZNF473 domains; P: promoter; TasR: tandem interspaced guide RNA (TIGR) associated RuvC nuclease; TnpB: transposon-encoded nuclease B.

One of the most promising therapeutic advantages of CRISPRa is its ability to activate endogenous paralogous genes that can compensate for loss-of-function mutations in disease-causing genes (Riedmayr et al., 2022). In conditions such as RP, for example, biochemical studies have shown that the PDE6A and PDE6B subunits possess enzymatic equivalence (Muradov et al., 2010). Based on this information, we hypothesized that upregulating the endogenous Pde6b gene using a dCasMINI-based CRISPRa system could compensate for Pde6a deficiency and restore photoreceptor function in an ENU-induced *Pde6a*-mutant mouse model (Wang et al., 2024; **Figure 1A**). In this model, a missense mutation in *Pde6a* results in a loss of phosphodiesterase activity that causes early photoreceptor degeneration.

To test this strategy, we first used an *in vitro* screening of single-guide RNAs (sgRNAs) targeting the *Pde6b* promoter to determine that sgRNA7 has the highest activation efficiency, leading to a significant increase in PDE6B expression at both the mRNA and protein levels. Robust transcriptional activation by CRISPRa enhanced PDE6B expression *in vitro* and *in vivo*. Following sgRNA optimization, we engineered an all-in-one AAV2.NN vector encoding dCasMINI fused to a miniaturized VPR activator (miniVPR), driven by the EFS promoter (**Figure 1A**). Intravitreal injection of this vector into *Pde6a*-mutant mice produced an approximately 10-fold increase in *Pde6b* mRNA levels *in vivo*. Western blot and immunofluorescence analyses further confirmed the strong expression of dCasMINI and a corresponding elevation of PDE6B protein in retinal tissues, with efficient *in vivo* delivery and functional activation of the target gene.

To evaluate the therapeutic efficacy of this dCasMINI-based gene activation strategy, we conducted a comprehensive series of structural and functional assessments of the retina. Optical coherence tomography imaging revealed that outer nuclear layer thickness was preserved in treated eyes, indicating protection against photoreceptor degeneration (**Figure 1A**). Correspondingly, electroretinography demonstrated significantly enhanced a-wave and b-wave amplitudes in treated mice, consistent with improved rod photoreceptor function. Behavioral assays provided further functional validation: optokinetic tracking revealed increased visual acuity in dCasMINI-treated animals, and looming-induced escape response tests showed enhanced visual defensive behavior with shortened response latencies compared to untreated controls.

These findings offer strong *in vivo* evidence that dCasMINI-mediated activation of *Pde6b* can partially compensate for *Pde6a* loss, leading to both structural preservation and functional improvement in a RP mouse model. This work establishes a proof of concept for the therapeutic benefit of using CRISPRa platforms to activate endogenous genes *in vivo*.

An important consideration for promoter-targeted CRISPRa approaches is whether gene expression is restored to physiologic levels or enhanced beyond them, and the implications this has for long-term safety. In most cases, CRISPRa-mediated activation tends to restore expression toward physiologic thresholds, though moderate supraphysiological increases can occur depending on the potency of the gRNA and activator domains. In our study, for example, *Pde6b* activation produced an approximately 10-fold increase in mRNA expression, which was sufficient to compensate for *Pde6a* loss and improve retinal function without detectable adverse effects. Notably, because CRISPRa recruits transcriptional machinery within the endogenous chromatin context, the resulting expression remains under native regulatory control, which may reduce the risk of ectopic or unregulated overexpression observed with exogenous cDNA supplementation. Nevertheless, sustained supraphysiological activation could potentially lead to cellular stress or dysregulation. Therefore, future long-term studies in chronic disease models will be essential to fully evaluate the safety profile of CRISPRa-based gene activation therapies.

CRISPRa-based strategies may be particularly advantageous in several disease contexts. First, they are well-suited for haploinsufficiency disorders, where partial restoration of gene activity is sufficient to provide therapeutic benefit. In addition, CRISPRa enables compensatory activation of paralogous genes to restore the function of a mutated gene, as demonstrated in our study by activating PDE6B to compensate for PDE6A deficiency in retinitis pigmentosa. Second, CRISPRa provides a unique opportunity for conditions involving large genes such as ABCA4, MYO7A, and USH2A, whose cDNA sequences exceed the packaging capacity of AAV vectors. Rather than directly supplementing these oversized genes, CRISPRa can upregulate functionally redundant or related endogenous genes. Third, in the context of neural regeneration, CRISPRa allows coordinated upregulation of multiple transcription factors and neuroprotective genes, which may be required for promoting survival, reprogramming, or axonal regeneration in retinal and central nervous system disorders. Together, these features underscore the versatility of CRISPRa as a therapeutic platform across diverse genetic and neurodegenerative diseases.

While our study demonstrates the feasibility and therapeutic efficacy of *in vivo* gene activation using an all-in-one dCasMINI-miniVPR AAV system, several challenges remain. Notably, the total size of the construct, consisting of the dCasMINI-miniVPR fusion protein, promoter, and sgRNA cassette, approaches the upper limit of packaging capacity of AAV. This constrains the flexibility of the system, particularly for applications requiring multiple sgRNAs to activate complex gene networks. Although dCasMINI performs well *in vitro* and *in vivo*, its activation efficiency and guide RNA compatibility are less than those of the established SpCas9-based CRISPRa systems.

To enhance the potential of CRISPRa for clinical gene therapy, the development of next-generation transcriptional activators that are smaller, more efficient, and more versatile will be essential, particularly within the constraints of clinically viable delivery vehicles such as AAV. Looking ahead, we propose three key areas for optimization to advance CRISPRa-based therapeutics (**Figure 1B**).

First is further miniaturization of CRISPRa components. Reducing the total payload size will allow inclusion of additional regulatory elements, such as multiple sgRNAs, which will enable complex gene activation programs from a single AAV vector. Recent developments in ultra-compact genome editors provide additional opportunities for building miniaturized CRISPRa systems. In addition to dCasMINI, emerging nucleases such as TnpB, AsCas12f1, and TasR offer even smaller coding sequences (300–500 amino acids) and distinct PAM requirements, enabling more efficient packaging into AAV vectors (Hino et al., 2023; Faure et al., 2025). These compact nucleases can be fused with miniaturized activation domains (e.g., miniVPR, NFZ, and MSN) to create highly flexible and potent transcriptional activators suitable for *in vivo* applications. While dCasMINI demonstrates robust activation in retinal tissues, these newer platforms provide alternative strategies to overcome size constraints and expand the therapeutic potential of CRISPRa for complex genetic and neurodegenerative disorders.

Second, enhancing transcriptional activation efficiency remains a top priority. Many compact Cas proteins exhibit lower baseline activity than SpCas9, likely reflecting a trade-off between size and DNA-binding strength (Faure et al., 2025). However, recent analyses in protein engineering offer several strategies to improve performance, including introducing point mutations to enhance DNA binding affinity, optimizing sgRNA structures to improve expression and target engagement, and fusing auxiliary DNA-binding domains to stabilize chromatin interactions. Integration of these approaches with compact Cas backbones could yield compact yet highly potent activators suitable for therapeutic use.

Third, enabling robust multiplexed gene activation will be essential, particularly for ocular applications requiring coordinated regulation of multiple genes. For example, vision restoration strategies via neuronal reprogramming often rely on simultaneous activation of multiple transcription factors, while enhancing retinal ganglion cell survival and axonal regeneration may necessitate co-expression of multiple protective genes (Soucy et al., 2023). To enable such complex interventions within the limited AAV capacity, compact guide RNA expression strategies such as tRNA- or ribozyme-based sgRNA arrays offer a powerful solution (Yuan and Gao, 2022). These self-cleaving RNA systems allow multiple sgRNAs to be transcribed from a single promoter, enabling efficient multigene regulation from a streamlined cassette. Together, these innovations in miniaturization, potency, and multiplexing have the potential to unlock the full therapeutic promise of CRISPRa, particularly for the treatment of complex and currently untreatable forms of inherited retinal disease.

In summary, our study establishes proof-of-concept that the miniaturized dCasMINI platform can effectively activate endogenous gene expression *in vivo* and restore visual function in a mouse model of RP. This strategy offers a compelling alternative to conventional gene supplementation, particularly for disorders involving large or structurally complex genes that exceed AAV packaging limits. Continued development of smaller, more potent, and multiplex-compatible CRISPRa tools will be critical for broadening the therapeutic approaches for gene activation. These innovations will not only improve delivery efficiency within AAV constraints but also enable modulation of gene networks across a wide range of inherited ocular diseases and potentially for genetic conditions affecting organs other than the eye.

*This work was supported by R01-EY025295, R01-EY032159, VA CX 001481, VA Merit (BX-006638) (all to YS), Children’s Health Research Institute Award (to YS); Research for Prevention of Blindness Unrestricted grant (Stanford Ophthalmology); International Retinal Research Foundation (to ZL); P30 NIH grant (Stanford Ophthalmology).*
